# Elevated cellular cholesterol in Familial Alzheimer’s presenilin 1 mutation is associated with lipid raft localization of β-amyloid precursor protein

**DOI:** 10.1371/journal.pone.0210535

**Published:** 2019-01-25

**Authors:** Yoon Young Cho, Oh-Hoon Kwon, Myoung Kyu Park, Tae-Wan Kim, Sungkwon Chung

**Affiliations:** 1 Department of Physiology, Sungkyunkwan University School of Medicine, Suwon, South Korea; 2 Department of Pathology and Cell Biology, and Taub Institute for Research on Alzheimer’s Disease and the Aging Brain, Columbia University Medical Center, New York, New York, United States of America; Torrey Pines Institute for Molecular Studies, UNITED STATES

## Abstract

Familial Alzheimer’s disease (FAD)-associated presenilin 1 (PS1) serves as a catalytic subunit of γ-secretase complex, which mediates the proteolytic liberation of β-amyloid (Aβ) from β-amyloid precursor protein (APP). In addition to its proteolytic role, PS1 is involved in non-proteolytic functions such as protein trafficking and ion channel regulation. Furthermore, postmortem AD brains as well as AD patients showed dysregulation of cholesterol metabolism. Since cholesterol has been implicated in regulating Aβ production, we investigated whether the FAD PS1-associated cholesterol elevation could influence APP processing. We found that in CHO cells stably expressing FAD-associated PS1 ΔE9, total cholesterol levels are elevated compared to cells expressing wild-type PS1. We also found that localization of APP in cholesterol-enriched lipid rafts is substantially increased in the mutant cells. Reducing the cholesterol levels by either methyl-β-cyclodextrin or an inhibitor of CYP51, an enzyme mediating the elevated cholesterol in PS1 ΔE9-expressing cells, significantly reduced lipid raft-associated APP. In contrast, exogenous cholesterol increased lipid raft-associated APP. These data suggest that in the FAD PS1 ΔE9 cells, the elevated cellular cholesterol level contributes to the altered APP processing by increasing APP localized in lipid rafts.

## Introduction

Alzheimer’s disease (AD) is characterized by the accumulation of β-amyloid peptide (Aβ) and the formation of neurofibrillary tangles in the brain; the highly amyloidogenic 42-residue Aβ (Aβ42) is the first species to be deposited in both sporadic and familial AD (FAD). Aβ42 and 40-residue Aβ (Aβ40) are produced by the interplay between β-amyloid precursor protein (APP) and the key enzymes. APP is first cleaved at its amino terminus of Aβ sequence by an aspartyl protease β-site-APP-cleaving enzyme (BACE1, or β-secretase), releasing a large N-terminal truncated APP (sAPPβ) and a membrane-associated carboxy terminal fragment (CTFβ, or C99) [1, 2]. The remaining CTFβ is cleaved by a γ-secretase complex that comprises presenilin 1 (PS1), presenilin enhancer 2 (PEN-2), nicastrin, and anterior pharynx defective 1 (APH1). This sequential cleavage produces Aβ40 and Aβ42 as well as APP intracellular domain (AICD) [3–5]. Alternatively, APP can be cleaved by α-secretase followed by γ-secretase, precluding Aβ production. It was initially established that mutations in FAD-associated PS1 and PS2 increase the ratio of Aβ42 to Aβ40 both in vivo and in vitro as well as in AD patients [6–9], although recent studies indicate that the increase in Aβ42 levels relative to Aβ40 may not be a universal phenotype associated with presenilin FADs [10]. Much evidence shows that cholesterol plays an important role in the pathogenesis of AD. Changes in lipid composition including increased cholesterol level are observed in AD patients and in postmortem AD brains [11, 12]. Most brain regions in AD patients show significantly increased cholesterol levels, and the intermediates involved in cholesterol biosynthesis such as lanosterol, demosterol, 24-hyroxycholesterol, and 27-hydroxycholesterol are also altered in AD patients and transgenic AD mouse model [13, 14]. Furthermore, APP processing and Aβ generation are influenced by cholesterol, likely because of the localization of the catalytic core of γ-secretase complex within the transmembrane domain [15–17].

Lipid rafts are membrane domains enriched in cholesterol and sphingolipids, which serve as platforms for protein-protein interactions and for cellular signaling [18–20]. It has been reported that β-secretase is localized in lipid raft microdomains by cysteine palmitoylation [21], and the four components of γ-secretase complex are also located in lipid rafts [16, 17]. In contrast, α-secretase is located in the non-raft domains. Furthermore, it has been demonstrated that β-secretase and APP are cross-linked together in lipid rafts by antibody co-patching system [22]. These findings indicate that APP may follow the amyloidogenic pathway predominantly in lipid rafts, whereas the non-amyloidogenic processing of APP occurs in the phospholipid-rich domain of the plasma membrane [23]. The link between cholesterol and Aβ generation has been shown in Niemann Pick type C disease model cells [24]. The authors showed that the increased cholesterol induced the shift of the overexpressed APP into lipid rafts, which suggests that cholesterol may regulate APP processing and play an important role in Aβ production.

Growing evidence indicates that presenilins (PSs) play several non-catalytic functions, including protein trafficking, ion channel regulation and autophagy [25–27]. In addition, it is reported that PSs are indispensable for maintaining membrane lipid composition. We have previously shown that PS mutations reduced phosphatidylinositol 4,5-bisphosphate (PI(4,5)P2) [28]. The ganglioside GM1 and cholesterol are increased and membrane fluidity is decreased in PS1-deficient mouse embryonic fibroblast cells [29]. When γ-secretase function is impaired, cholesterol is elevated in the plasma membrane as a result of decreased endocytosis of low-density lipoprotein (LDL) receptor [30]. This study showed that the increased cholesterol level could be explained by increased the mRNA level of lanosterol 14 alpha-demethylase (CYP51), an enzyme of cytochrome P450 super family, in FAD-associated PS1/PS2 deficient cells. CYP51 produces the intermediates of cholesterol synthesis from lanosterol. It converts 14α-methyl group into 14α-carboxyaldehyde and eliminates formic acid with the formation of the Δ14, 15 double bonds concomitantly [31, 32]. Furthermore, FAD-associated PS mutations in mouse embryonic fibroblasts (MEF) cells showed the perturbation of lipid homeostasis including increased cellular cholesterol [33]. From the close relationship between cholesterol and Aβ generation, it can be hypothesized that the elevated cholesterol level may influence APP processing. However, functional consequences of the cholesterol elevation associated with FAD PS1 are not known yet. Here we confirmed that cellular cholesterol level was elevated in FAD PS1 ΔE9 cells. We showed that in these cells the ratio of APP localized in lipid rafts was increased. We also demonstrated that the reduction of cellular cholesterol in PS1 ΔE9 cells not only reduced APP level in lipid rafts but decreased secreted Aβ42 levels. These results suggest that the increased cellular cholesterol level and subsequent enhancement of APP localization in lipid rafts is a cellular phenotype associated with FAD PS1 and may contribute to the altered APP processing and Aβ generation in these cells. Thus, in addition to a predisposition to AD, the high cholesterol level may contribute to the pathology of a genetic basis of AD.

## Results

### Cholesterol levels were elevated in CHO PS1 ΔE9 cells

**Fig 1:**
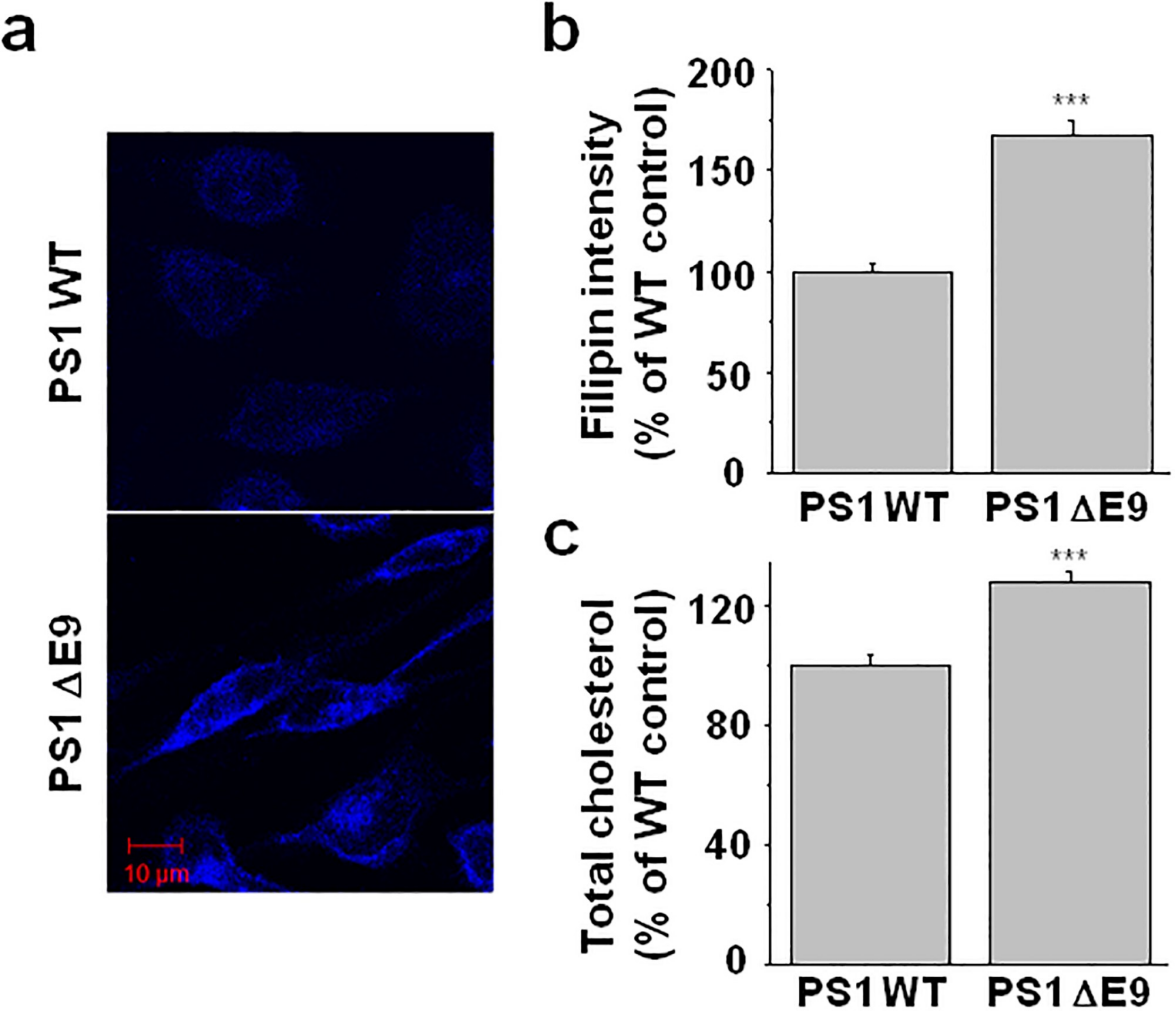
Cholesterol level was higher in the CHO PS1 ΔE9 cells than in the PS1 WT cells. (**a**) Free cholesterol was visualized by filipin staining from the CHO PS1 WT and ΔE9 cells. (**b**) Fluorescence intensities were compared between the PS1 WT (n = 11) and ΔE9 cells (n = 13). (**c**) Total cholesterol levels were measured from cell membrane fractions using the Amplex Red cholesterol assay kit from the PS1 WT and ΔE9 cells (n = 7). Statistical analysis was carried out using one way ANOVA: ***p<0.001.

It was shown that the increased expression of CYP51, which converts lanosterol into cholesterol, underlies the elevated cholesterol levels with FAD-associated PS1/PS2 deficient MEF cells [30]. In addition, a previous report showed that FAD-associated PS mutations showed increased cholesterol in MEF cells [33]. Using two complementary experimental approaches, we found that cholesterol was elevated in the CHO cells stably expressing the PS1 ΔE9 mutant. First, we incubated PS1 WT and ΔE9 cells with 0.05 mg/ml filipin for 2 h. Since filipin specifically binds to free cholesterol resulting in a fluorescent signal, we visualized the free cholesterol levels under fluorescence microscopy (Fig 1a). Compared with the PS1 WT cells, fluorescence intensity was elevated in PS1 ΔE9 cells by 67.6 ± 6.7% (n = 13; Fig 1b). We next used a fluorometric method to measure total membrane cholesterol. Cholesterol was significantly higher in the CHO PS1 ΔE9 cells (by 27.7 ± 3.58%, n = 4) compared with the PS1 WT cells (Fig 1c), which was consistent with the results from the filipin staining. Western blot analysis showed that CYP51 expression levels were higher by 25.3 ± 0.17% (n = 6) in the CHO PS1 ΔE9 cells compared with the PS1 WT cells (S1 Fig). Thus, the increased CYP51 expression may underlie the elevated cholesterol level in PS1 ΔE9 cells. This result is consistent with a previous report [30].

### APP localized in lipid rafts increased in CHO PS1 ΔE9 cells

A large body of evidence indicates that amyloidogenic processing of APP occurs in lipid rafts given that the subset of β-, γ- secretases and APP are localized in lipid raft microdomains [16, 17, 22, 23, 34, 35]. Not only is cholesterol considered the determinant factor for lipid rafts structure, but cholesterol binding to APP/C99 is suggested as a working model for how cholesterol is linked to amyloidogenic processing [36–38]. From these previous results, we hypothesized that the elevated cholesterol in PS1 ΔE9 cells may directly influence the localization of APP in lipid rafts. To test this idea, we isolated lipid rafts using two commonly used non-ionic detergents, Triton X-100 and Brij-98. The PS1 WT and ΔE9 cells were homogenized and lysates were sonicated in the presence of either Triton X-100 or Brij-98, which was followed by loading equal amount of protein on discontinuous sucrose gradients [39]. After obtaining 12 fractions, equal volumes of each fraction was loaded on SDS gels to detect APP and caveolin, a marker for lipid rafts. We could not detect APP in lipid raft fractions with the use of Triton X-100 (S2 Fig). When we used Brij-98, APP localized in lipid raft fractions was detectable only with long exposure.

**Fig 2:**
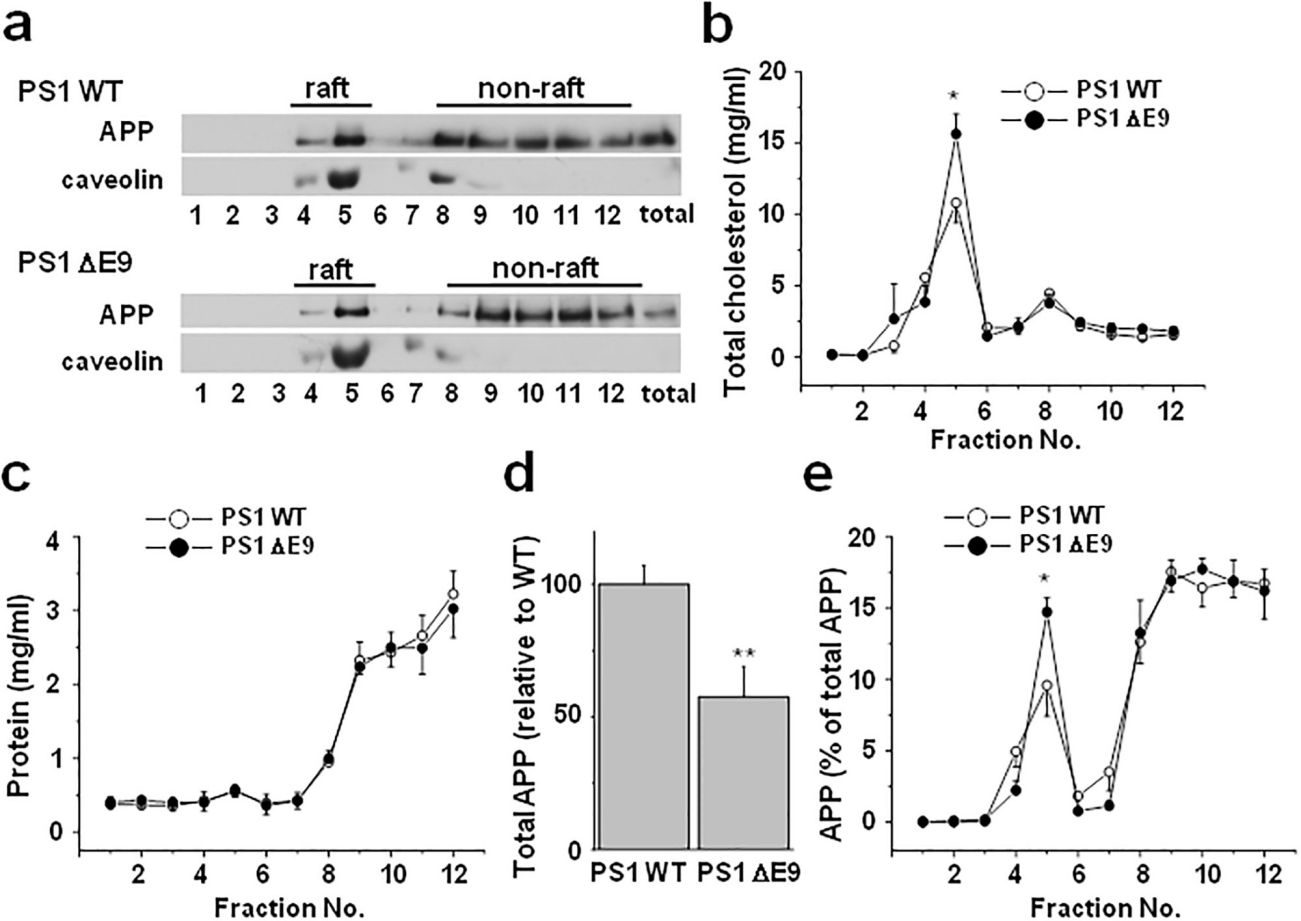
The ratio of APP localized in lipid rafts was higher in the CHO PS1 ΔE9 cells than in the CHO PS1 WT cells. The CHO PS1 WT and ΔE9 cells were homogenized with sodium carbonate buffer as described in Methods. The cell lysates were used for discontinuous sucrose density gradients. The gradients were harvested from top to bottom to obtain 12 fractions, and equal volumes of each fraction were loaded on SDS gels. (**a**) A representative western blot shows the APP and caveolin (lipid raft marker) expression levels in the CHO PS1 WT and ΔE9 cells. (**b**) Analysis of cholesterol levels and (**c**) protein levels from sucrose gradient fractions. In each fraction, cholesterol levels were measured using Amplex Red cholesterol assay kit (n = 4), and protein levels were determined using BioRad protein assay (n = 4). PS1 ΔE9 cells showed higher cholesterol levels in lipid raft fractions compared to CHO PS1 WT cells. (**d**) The densitometric analysis of total APP from cell lysates of CHO PS1 WT and ΔE9 cells (n = 7). Note that CHO PS1 ΔE9 cells showed decreased APP levels than CHO PS1 WT cells. (**e**) The densitometric analysis of the ratio of APP in each fraction (n = 5). The ratio of APP localized in lipid raft fractions was higher in the PS1 ΔE9 cells than in the PS1 WT cells. One-way ANOVA. *p<0.05.

Since it is possible that the presence of detergents could be the reason for not detecting APP in lipid raft fractions, we isolated lipid rafts using the experimental method that does not involve detergent extraction shown in Fig 2a. The low density and caveolin-rich lipid raft fractions showed high cholesterol levels, while the high density non-raft fractions were lower in cholesterol levels (Fig 2b). Interestingly, cholesterol levels in non-raft fractions did not differ between the PS1 WT and ΔE9 cells, while it was higher only in lipid raft fractions in the PS1 ΔE9 cells compared to the PS1 WT cells. Thus, most of the elevated cholesterol in the PS1 ΔE9 cells could be attributed to the elevated cholesterol levels in lipid rafts. Caveolin level in lipid raft fraction was also increased in PS1 ΔE9 cells compared to PS1 WT cells. Most of the proteins were localized in non-raft fractions, and the protein levels in each fraction did not differ between the PS1 WT and ΔE9 cells (Fig 2c). This clear separation between protein-high non-raft fractions and cholesterol-high lipid raft fractions indicated that the fractionation of lipid rafts and non-rafts was well established [40]. The APP levels in 12 fractions from the PS1 ΔE9 cells were lower than the levels from the PS1 WT cells (Fig 2a). Similarly, total APP level in the cell lysate was lower in ΔE9 cells than the levels in the PS1 WT cells as shown at the last lane in Fig 2a. The densitometric analysis of total APP levels in lysate is shown in Fig 2d. When we calculated the ratio of APP in each fraction, the majority of APP was localized in non-raft fractions (Fig 2e). However, a sizable amount of APP was co-fractionated with lipid raft fractions. The most noticeable difference between PS1 WT and ΔE9 cells was the ratio of APP localized in lipid rafts even though ΔE9 cells shows lower total APP compared to WT cells. This is in line with the elevated cholesterol level only in ΔE9 cells’ lipid raft fractions compared to WT cells. The percentage of APP localized in lipid raft fractions was significantly higher in CHO PS1 ΔE9 cells than in PS1 WT cells. Similar result was observed when we separately combined the lipid raft fractions (#4 and #5) and non-raft fractions (from #8 to #12), and equal amount of proteins was loaded for western blot (S3 Fig). Our results suggest that the elevated cholesterol in PS1 ΔE9 cells may promote the association of APP with lipid rafts.

Then, we measured the levels of α-secretases (ADAM 9, ADAM10, ADAM17), β-secretase (BACE-1), and a component of γ-secretase (Nicastrin) from both lipid raft and non-raft fractions by loading equal amount of protein for western blot. They were not different between PS1 WT and PS1 ΔE9 cells (S4 Fig).

### γ-secretase inhibitor failed to change lipid rafts localization of APP in PS1 ΔE9 cells

It has been proposed that PS mutations affect γ-secretase function in view of presenilin as a catalytic core of γ-secretase. We could hypothesize that APP localization into lipid rafts by cholesterol elevation in FAD-associated PS1 ΔE9 cells could be the additional effect of impaired γ-secretase activity. To determine whether the increased APP localization in the lipid rafts from PS1 ΔE9 cells was related to altered γ-secretase activity in PS1 ΔE9 cells, we tested the effect of the potent, widely used γ-secretase inhibitor (S5 Fig). Treating the PS1 ΔE9 cells with 0.5 μM γ-secretase inhibitor IX (Millipore, 565770) for 24 h did not produce any significant effects on APP localization in the lipid rafts, indicating that the FAD PS1 ΔE9-associated APP localization change in lipid rafts occurs independently from the catalytic function of PS1.

### Changes in cholesterol level altered the APP localization in lipid rafts

**Fig 3:**
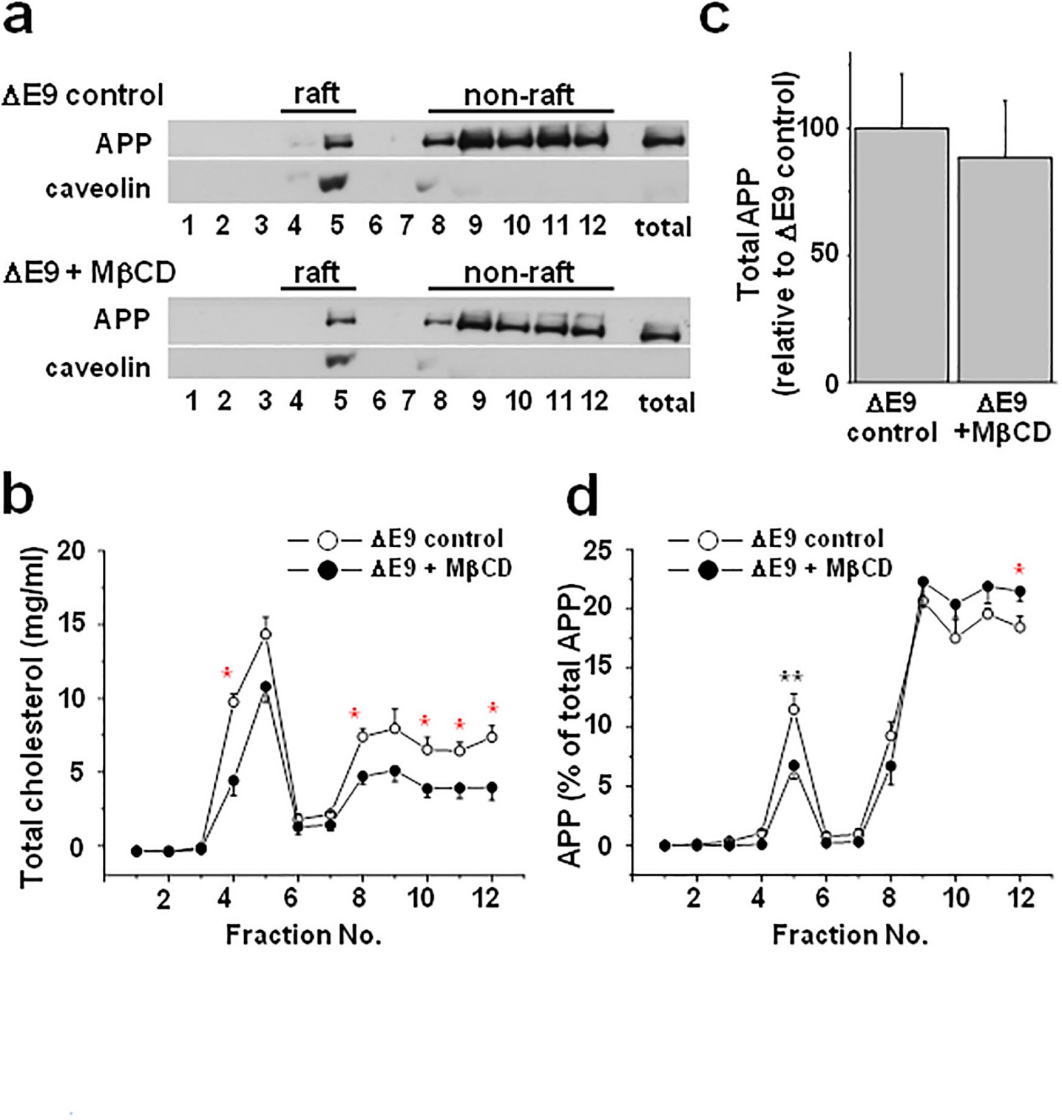
Reduction of cholesterol levels by MβCD decreased APP localization in lipid rafts from CHO PS1 ΔE9 cells. CHO PS1 ΔE9 cells were treated with or without 5 mM MβCD for 30 min followed by discontinuous sucrose density gradient to obtain lipid raft and non-raft fractions. (**a**) A representative western blot shows the APP and caveolin (lipid raft marker) expression levels from 12 fractions. (**b**) Analysis of cholesterol levels from the sucrose gradient fractions (n = 4). (**c**) The densitometric analysis of total APP from cell lysates with or without 5 mM MβCD treatment (n = 3). (**d**) The densitometric analysis of the ratio of APP levels in each fraction (n = 5). MβCD reduced the ratio of APP localization in lipid rafts. One-way ANOVA: *p<0.05, **p<0.01.

To test the effect of cholesterol reduction on the localization of APP in lipid rafts from the PS1 ΔE9 cells, we used methyl-β-cyclodextrin (MβCD) to lower membrane cholesterol levels. MβCD is commonly used to extract membrane cholesterol in cellular systems, although the amount of extracted cholesterol varies. The cells were incubated with 5 mM MβCD for 30 min. Under this condition, the cholesterol level was reduced by 29% (S6 Fig). Cholesterol extraction reduced APP and caveolin levels in lipid rafts as shown in Fig 3a. Extracting cholesterol using MβCD reduced cholesterol levels from both non-raft and raft fractions (Fig 3b), indicating that cholesterol-lowering effect of MβCD is not selective to lipid rafts. The total APP from cell lysates was not changed by 5 mM MβCD treatment as shown at the last lane in Fig 3a, and by the densitometric analysis of total APP levels in Fig 3c. However, the ratio of APP localized in lipid rafts significantly decreased after cholesterol extraction using MβCD (Fig 3d). The ratio of APP localized in non-rafts was increased by MβCD, although it was not statistically significant. From these results, cholesterol reduction by MβCD, which affected cholesterol in both lipid raft and non-raft fractions, decreased the ratio of APP localization in lipid rafts.

Conversely, we tested whether increasing cholesterol level increased the ratio of APP localization in lipid rafts from CHO PS1 WT cells. Cells were treated for 1.5 h with 75 μM MβCD saturated with cholesterol (MβCD-cholesterol), an agent which is commonly used for adding cholesterol exogenously. We found that addition of exogenous cholesterol lead to the increase of the ratio of APP localization in lipid rafts from PS1 WT cells (S7 Fig). Our results suggest that elevating cholesterol levels in the PS1 WT cells recapitulated the increased APP localization in lipid rafts in PS1 ΔE9 cells.

To confirm whether the cholesterol level affected endogenous APP localization in lipid rafts from neuronal cells, we used human neuroblastoma SH-SY5Y cells. We obtained lipid raft and non-lipid raft fractionations from cells and then, we collected fraction #4 and #5 as lipid raft fractions and fraction #8 to #12 as non-raft fractions for western blotting. Equal amount of proteins were loaded to observe endogenous APP distribution in lipid raft and non-raft fractions. Endogenous APP was barely detectable in either non-raft or raft fractions in human SH-SY5Y cells with 48 h exposure (S8 Fig). Because the endogenous APP expression was undetectable using western blotting, the SH-SY5Y cells were stably transfected with APP and BACE1 (SH-SY5Y-APP/BACE1). To enrich the cholesterol levels, the SH-SY5Y-APP/BACE1 cells were treated with 75 μM MβCD-cholesterol for 30 min. After lipid raft fractions were isolated, we detected APP and flotillin-1, another lipid raft marker (S9 Fig). Densitometric analysis of the western blots showed that the ratio of APP localized in lipid rafts significantly increased after the MβCD-cholesterol treatment. Cholesterol levels were higher in non-raft fractions as well as lipid raft fractions, indicating an overall increase in cellular cholesterol by MβCD-cholesterol. Total protein levels were not changed by MβCD-cholesterol. These results indicate that the elevated cholesterol level in the neuronal model cells increased APP localization in lipid rafts.

### Tebuconazole, a CYP51 inhibitor, reversed the elevated cholesterol level as well as increased APP distribution into lipid rafts in PS1 ΔE9 cells

**Fig 4:**
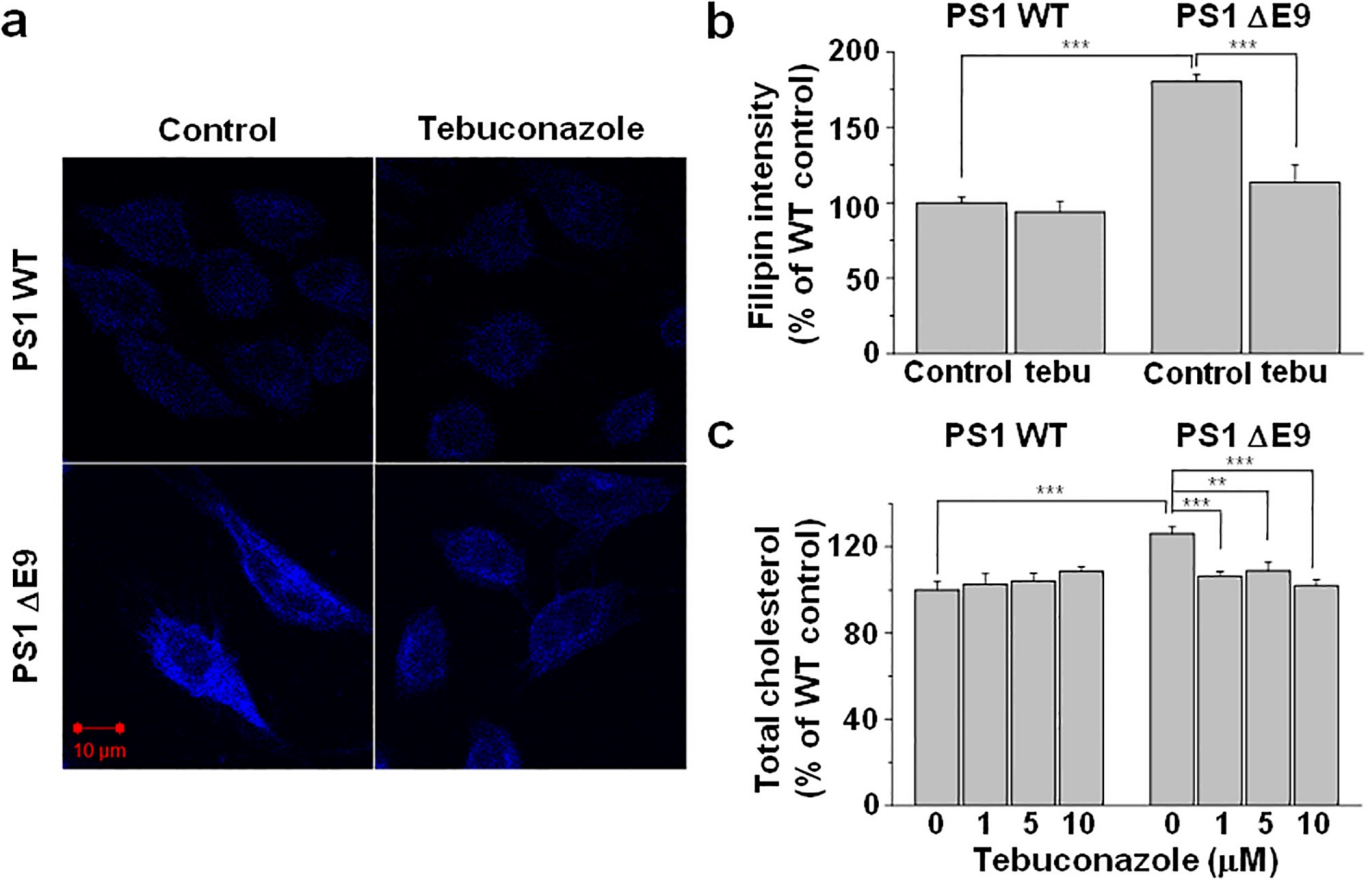
Elevated cholesterol level in CHO PS1 ΔE9 cells was decreased by the inhibition of cholesterol biosynthesis. (**a**) CHO PS1 WT and ΔE9 cells were pre-treated with 1 μM tebuconazole for 48 h, and free cholesterol was visualized by filipin staining. (**b**) Filipin intensities were compared in the absence (control) and presence of tebuconazole (tebu). PS1 WT control (n = 14), PS1 WT with 10 μM tebuconazole (n = 11), ΔE9 control (n = 10), ΔE9 with 10 μM tebuconazole (n = 10). (**c**) CHO PS1 WT and ΔE9 cells were pre-treated with 0, 1, 5, or 10 μM tebuconazole for 48 h, and total cholesterol level was measured from cell membrane fractions using Amplex Red cholesterol assay kit (n = 8). One way ANOVA: **p<0.01, ***p<0.001.

Given the reported role for CYP51 in the elevated cholesterol levels in the FAD PS1 cells [30], we next tested whether the increased expression of CYP51 underlies the elevated cholesterol level in PS1 ΔE9 cells. For this purpose, we utilized tebuconazole, which is a specific inhibitor for CYP51. Cells were treated with 10 μM tebuconazole for 48 h. Filipin fluorescence intensity from PS1 ΔE9 cells was decreased to a comparable level of control PS1 WT cells by tebuconazole (Figs 4a and 4b). Similarly, total membrane cholesterol levels were decreased by tebuconazole from PS1 ΔE9 cells when fluorometric method was used (Fig 4c). However, tebuconazole exerted no apparent effects on the levels of cholesterol in the PS1 WT cells. These results may suggest that CYP51 inhibition by tebuconazole is selective to the cellular mechanism involving PS1 ΔE9 cells. It is possible that cells may increase CYP51 expression in the presence of its inhibitor as a compensatory mechanism. Indeed, when we examined CYP51 expression level using western blotting, it was increased in the presence of tebuconazole both in PS1 WT and ΔE9 cells (S10 Fig). However, further studied will be needed to fully understand the regulation of CYP51 expression, and the selective effect of tebuconazole on PS1 ΔE9 cells.

**Fig 5:**
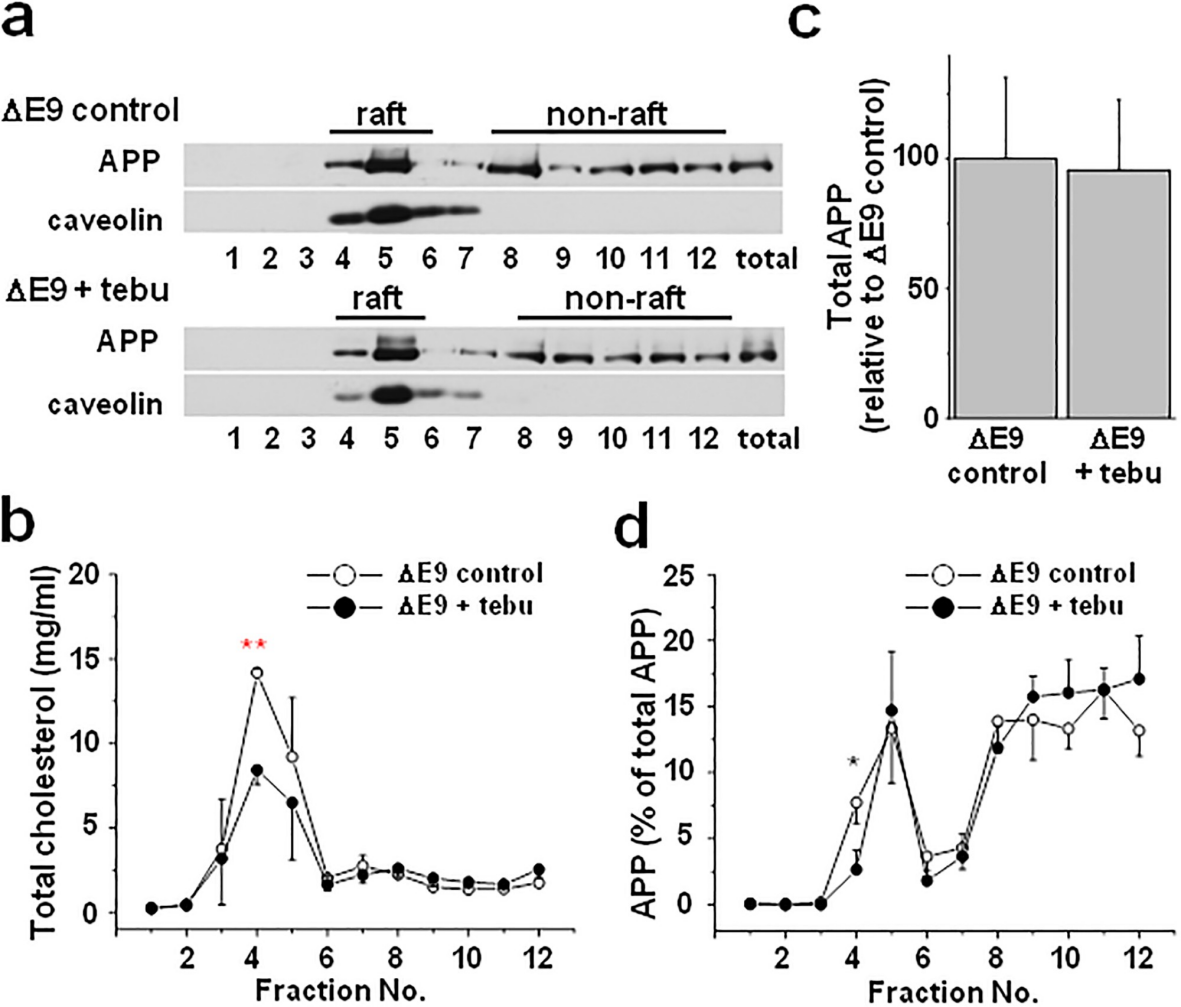
The ratio of APP localized in lipid rafts was decreased by reducing cholesterol levels using tebuconazole from CHO PS1 ΔE9 cells. CHO PS1 ΔE9 cells were pre-treated without (ΔE9 control) or with (ΔE9 + tebu) 10 μM tebuconazole for 48 h. Cell lysates were used for discontinuous sucrose density gradient to fractionate into raft and non-raft fractions. (**a**) APP and caveolin levels from 12 fractions were measured using western blots. (**b**) The levels of cholesterol in each fraction were analyzed using Amplex Red cholesterol assay kit (n = 3). Cholesterol levels in lipid raft fractions were decreased by tebuconazole. (**c**) The densitometric analysis of total APP from cell lysates with or without tebuconazole (n = 7). Total APP was not changed by tebuconazole. (**d**) The densitometric analysis of the ratio of APP levels in each fraction (n = 6). The ratio of APP in lipid raft fractions was decreased by tebuconazole consistent with cholesterol reduction by tebuconazole treatment. One-way ANOVA: *p<0.05, **p<0.01.

In the following experiments, we used tebuconazole as a tool to reduce the elevated cholesterol level associated with PS1 ΔE9 cells into a comparable level to PS1 WT cells. First, we tested whether the inhibition of CYP51 using tebuconazole could reverse the observed increased occurrence of APP in lipid rafts from the PS1 ΔE9 cells. Tebuconazole treatment significantly decreased levels of the caveolin levels consistent with the reduction of cholesterol levels in lipid raft fractions (Figs 5a and 5b). The total APP from cell lysates was not changed by tebuconazole treatment as shown at the last lane in Fig 5a, and by the densitometric analysis of total APP levels in Fig 5c. However, quantitative analysis revealed that the ratio of APP localized in lipid rafts was significantly decreased by tebuconazole treatment (Fig 5d). Then, we tested whether the presence of tebuconazole affected APP localization in lipid rafts from the PS1 WT cells (S11 Fig). Tebuconazole exerted no apparent effects on APP localization in lipid rafts from the PS1 WT cells, consistent with no effects of tebuconazole on the cellular cholesterol levels from these cells. In addition, tebuconazole exerted no effects on cholesterol level in non-raft fractions as well as lipid raft fractions from the PS1 WT cells. Together, these results suggest that the CYP51 inhibition can reverse the elevated levels of cholesterol and the increased lipid raft localization of APP in the PS1 ΔE9 cells.

### Tebuconazole decreases Aβ42 levels in PS1 ΔE9 cells but not in PS1 WT cells

**Fig 6:**
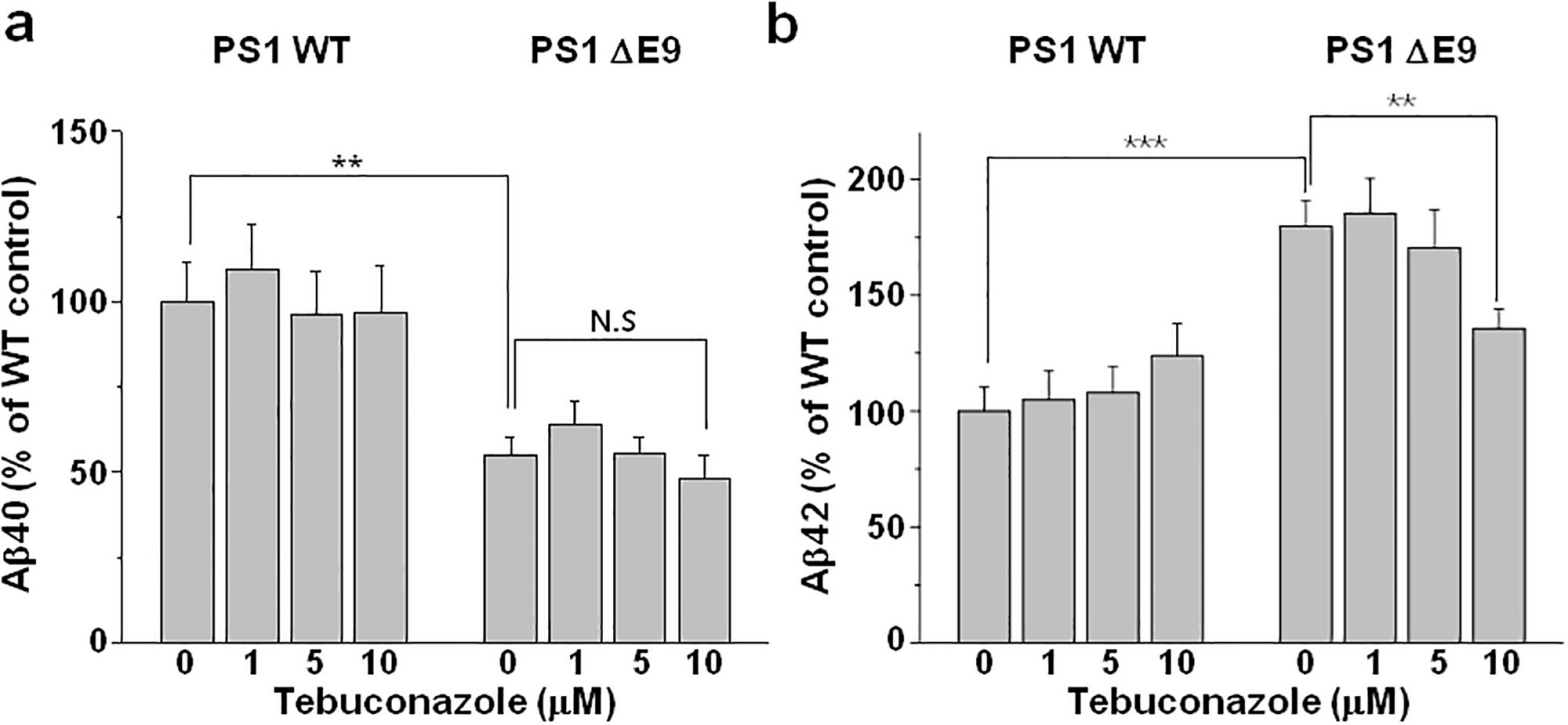
Tebuconazole decreased secreted Aβ42 levels in CHO PS1 ΔE9 cells but not in PS1 WT cells. CHO PS1 WT and ΔE9 cells were pre-treated with 0, 1, 5, or 10 μM tebuconazole for 48 h, and Aβ levels were measured from the conditioned media using (**a**) Aβ40 specific (n = 12) or (**b**) Aβ42 specific ELISA kit (n = 12). One-way ANOVA: *p<0.05, **p<0.01, ***p<0.001.

In most of FAD-associated PS mutants, secreted Aβ42/Aβ40 ratio increases [7–9]. To confirm this observation in ΔE9 mutant, levels of Aβ40 and Aβ42 were measured from the conditioned media from CHO PS1 WT and ΔE9 cells by using sandwich ELISA method. The secreted Aβ40 level in PS1 ΔE9 cells was lower than that of PS1 WT cells (Fig 6a). This can be explained by a recent finding that FAD mutants PS-1/γ-secretase complexes reduce carboxypeptidase activity while increasing the Aβ42/Aβ40 ratio [41]. Alternatively, the decreased Aβ40 level may be due to the reduced APP level in PS1 ΔE9 cells. In contrast, secreted Aβ42 levels in PS1 ΔE9 cells were significantly increased by 79.7 ± 11.1% compared to PS1 WT cells (Fig 6b, n = 12). Then, we examined the effects of tebuconazole on the secreted Aβ40 and Aβ42 levels. As shown in Fig 6a, tebuconazole reduced Aβ40 only from PS1 ΔE9 cells. However, the effects were not significant. Tebuconazole decreased Aβ42 levels in a dose-dependent manner from PS1 ΔE9 cells. At 10 μM tebuconazole, Aβ42 level was significantly decreased by 44%. In contrast to PS1 ΔE9 cells, PS1 WT cells showed no significant changes in Aβ42 levels by tebuconazole. At these tebuconazole concentrations, cell viability was not affected (data not shown). Thus, reducing the elevated cholesterol level in PS1 ΔE9 cells by tebuconazole treatment specifically decreased the secreted Aβ42 levels, which is one of cellular phenotypes associated with PS1 ΔE9. These results suggest that the elevated cholesterol level may be directly linked to the enhanced secreted Aβ42 level in PS1 ΔE9 cells.

## Discussion

Specific molecular phenotypes associated with FAD PS have long been under intense investigation. Owing to the PS's role as a catalytic subunit, it has been postulated that FAD-linked mutations in the PS genes universally affect the γ-secretase activity resulting in the increase of Aβ42 relative to Aβ40 (Aβ42/Aβ40 ratio) [41, 42]. However, recent studies revealed that the reconstituted γ-secretase complex harboring FAD mutant forms of PS1 do not overproduce Aβ42 or change the Aβ42/Aβ40 ratio in vitro, suggesting that additional cellular mechanisms may be involved with the altered γ-secretase activity observed in the FAD PS1 cells [10]. In addition to the catalytic function, PS1 is involved with several non-catalytic cellular functions, such as Ca^2+^ influx and protein trafficking, in a γ-secretase-independent manner [25–27].

**Fig 7:**
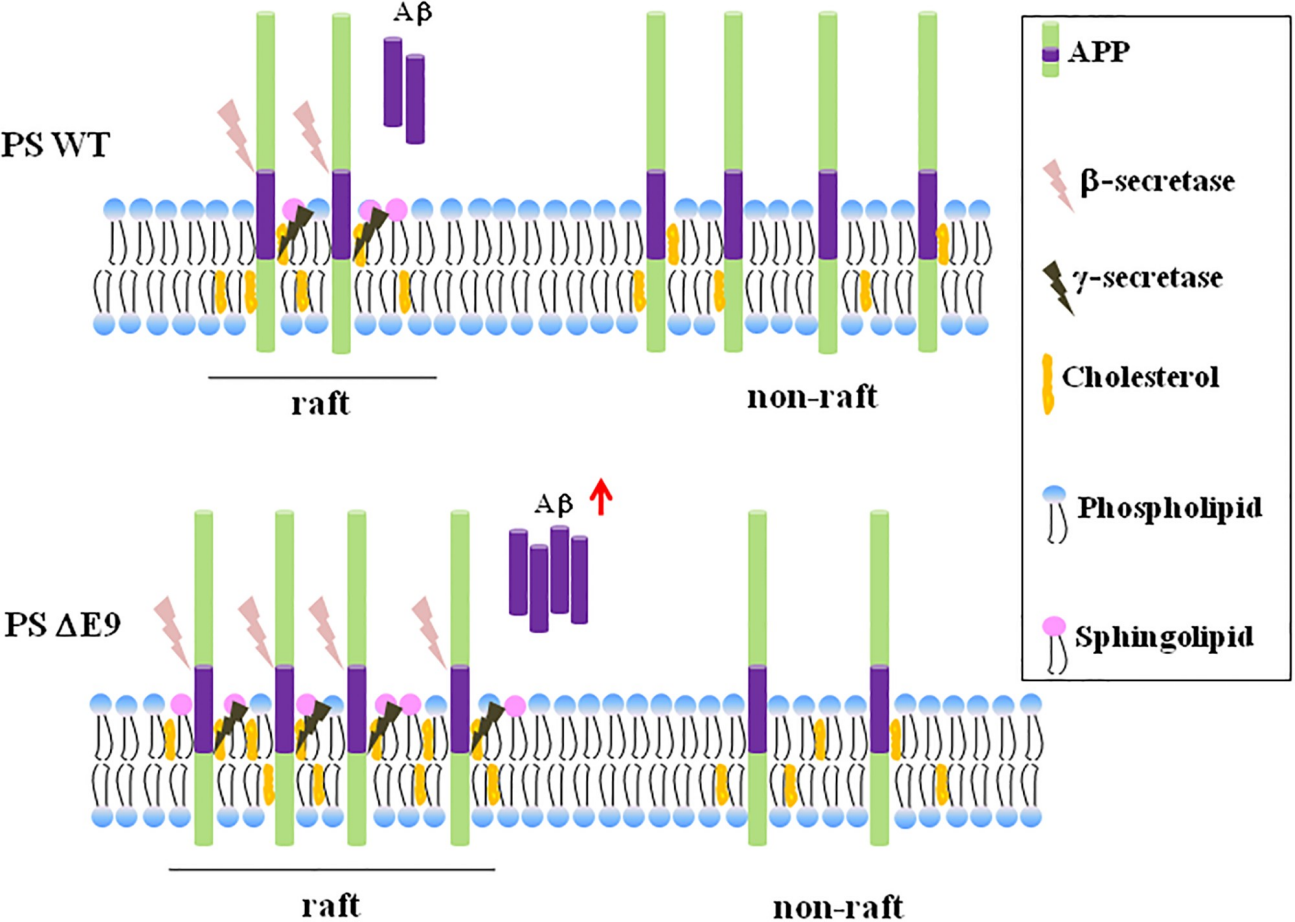
Our current model for the effects of PS1 mutant on APP localization in lipid rafts and Aβ production. Elevated cholesterol in PS1 mutant increases lipid rafts structures altering APP distribution into lipid rafts, which produces more Aβ42 peptides.

Cholesterol has been extensively implicated in the regulation of cellular APP processing [43–46]. We showed that the cholesterol level is higher in PS1 ΔE9 cells than in PS1 WT cells, which is consistent with a previous report that FAD-associated PS mutations in MEF cells showed increased cellular cholesterol [33]. In this study, we used CHO cells that were stably expressing APP. These cells were also stably transfected either with PS1 WT or with PS1 ΔE9. Because PS1 ΔE9-tansfected cells showed moderate but significant increase in cholesterol levels endogenously, we were able to rule out the effect of APP on cholesterol metabolism. The ratio of APP localized in lipid rafts was higher in the PS1 ΔE9 cells than in the PS1 WT cells. Reducing cholesterol using either MβCD or tebuconazole redistributed APP from lipid rafts into non-raft fractions in the PS1 ΔE9 cells. Tebuconazole also decreased the level of secreted Aβ42 from PS1 ΔE9 cells. Conversely, increasing cholesterol in the PS1 WT cells and in the neuronal model cells showed the opposite effect. These results may suggest that the impaired cholesterol homeostasis in PS FAD mutants is directly linked to the APP localization in lipid rafts, contributing to the increased Aβ42 secretion as shown in Fig 7 for our current model for the effects of elevated cholesterol on APP localization in lipid rafts and Aβ production. However, our results using model cells expressing APP may have limited physiological implication because the lipid composition and microdomain distribution in neuronal cells are different from these cells.

Lipid raft microdomains, a particular structure enriched with cholesterol and sphingolipids, are considered cellular processing platforms. With the antibody cross-linking method, it is reported that APP and β-secretase are co-patched in lipid rafts and that Aβ production increases as cholesterol levels increase [22]. According to FRET experiments, clustering of APP and β-secretase in lipid rafts on plasma membranes and intracellular compartments is triggered by exposure of cholesterol in primary neurons [47]. In addition, it has been demonstrated that the enlarged raft structure that results from increased cholesterol enhances APP’s access to its processing enzymes, β- and γ-secretases [22, 23, 34, 47]. Considering these previous results, it is possible that the increased cholesterol in PS1 ΔE9 cells may affect Aβ production by interacting with APP to alter its processing as suggested recently [36–38]. In this study, we isolated lipid raft domains using a biochemical method, and we showed direct evidence that the elevated cholesterol levels in PS1 ΔE9 cells changed the localization of APP in lipid rafts. Consistent with our conclusions, in model cells for Nieman Pick type C disease, the increased cholesterol level shifts APP into lipid rafts [24]. In our result, APP was not detectable in lipid rafts when we isolated them using the widely used non-ionic detergents Triton X-100 and Brij-98. It has been reported that lipid states in cellular membranes (gel state or liquid ordered state) as well as the kind of detergents and buffer system affected the solubilization of lipid raft domains [48]. Furthermore, the ‘sub-solubilizing’ stage which is the remaining large structure of lipids is not floated but easily submerged into heavier-density fractions when it is isolated with detergents [49], and the detergent-resistant membranes should not co-migrate with membrane rafts [50]. Furthermore, these detergents may cause the loss of lipid rafts components and/or detergent-induced non-specific aggregation [48–51]. In contrast, a sizable fraction of APP was localized in lipid rafts when we used non-detergent conditions with a sodium carbonate buffer. Because the estimated binding affinity between APP and cholesterol is very weak [52, 53], Triton X-100 or Brij-98 may disrupt the interaction between them and displace APP from lipid rafts.

Lipid dyshomeostasis, including elevated levels of cholesterol, plays a critical role in the pathogenesis of AD [54–57], but functional consequences of the cholesterol elevation associated with FAD PS1 is not known. Our current work showed that cholesterol increase occurring in lipid rafts of the PS1 ΔE9 cells is associated with the increased recruitment of APP into lipid rafts, and subsequently elevated the Aβ42 production. PS1 ΔE9 mutation is known to potentiate the Aβ42/Aβ40 ratio [41] and many different FAD PS mutations showed increased cellular cholesterol [33]. Thus, our results raise the novel possibility that the changes in cellular cholesterol levels associated with FAD PS may influence the localization of APP in lipid rafts. However, it is still unclear how the increased localization of APP in lipid rafts by cholesterol enrichment is directly linked to the enhanced Aβ production in the PS1 ΔE9 mutants. One possibility is that the increased plasma membrane cholesterol triggers clathrin-dependent APP endocytosis and increases Aβ secretion as shown in cultured cell lines [58]. Another possible link is that the increased cholesterol concentration near the active site of γ-secretase may affect carboxypeptidase activity. γ-secretase complex has carboxypeptidase activity that cleaves Aβ48 or Aβ49 residues to shorter secreted Aβ38 to Aβ43 residues by trimming the endoproteolytic ε and ζ sites of two long Aβ peptides [41]. PS1 mutations such as G384A, ΔE9, L166P, and A246E reduce the carboxypeptidase activity, and the endoproteolytic cleavage at the ε site decreases and Aβ42 increases [41, 42]. The other possibility is that since β- and γ-secretases are abundant in lipid rafts from AD model cell as well as human AD patients [15–17, 21, 22, 59–62]. The effect of increased cholesterol on APP localization in lipid rafts may promote access of β- and γ-secretase to APP. Consistent with this possibility, colocalization of APP and BACE in lipid rafts is increased in the entorhinal cortex of AD patients [63].

## Materials and methods

### Cell culture and experimental treatments

CHO cells stably expressing wild-type human APP751 were used [64]. Using these cells as parental cell lines, they were stably transfected with 10 μg of each plasmid of PS1 wild type (WT) and ΔE9 mutant using the SuperFect (Qiagen) transfection reagent according to the manufacturer’s protocol. Individual Zeocin-resistant colonies were isolated and screened for PS1 expression by western blotting. Levels of PS1 expression were not different between PS1 WT and PS1 ΔE9 cells as shown in S12 Fig. Stable cell lines were maintained in Dulbecco’s Modified Eagle Medium (DMEM) supplemented with 10% (v/v) heat-inactivated fetal bovine serum (FBS), 100 U/ml penicillin, 100 μg/ml streptomycin, and 250 μg/ml Zeocin and maintained at 37°C in an atmosphere containing 5% CO_2_. CHO PS1 WT and ΔE9 cells were incubated with either 10 μM tebuconazole 48 h. PS1 ΔE9 cells were treated with 5 mM MβCD for 30 min. CHO PS1 ΔE9 cells were incubated 0.5 μM γ-secretase inhibitor IX for 24 h. For the cholesterol replenishment experiment, CHO PS1 WT and SH-SY5Y-APP/BACE cells were treated with 75 μM MβCD-cholesterol for 1.5 h and 30 min, respectively.

### Filipin staining

We performed filipin (Sigma-Aldrich) staining to monitor the levels of free cholesterol from CHO PS1 WT and ΔE9 mutant cells. Cells were placed on poly-D-lysine-coated cover slips. The next day, the cells were washed three times with phosphate-buffered saline (PBS) for 5 min. Then, cells were fixed with 4% paraformaldehyde for 15 min at room temperature; the cells were washed with PBS three times. Then, cells were incubated with 1.5 mg/ml glycine in PBS for 10 min at room temperature to quench the paraformaldehyde. In the dark, cells were stained with 0.05 mg/ml filipin in PBS with 2% goat serum for 2 h at room temperature. We observed the fluorescence staining by confocal microscopy using a model LSM 710 microscope (Zeiss). We used the ImageJ program to quantify the images to measure mean fluorescence intensity in the region of interest according to the cell shape.

### Cell fractionation and cholesterol assay

Cells were washed twice with ice-cold PBS and lysed with hypotonic buffer (25 mM Tris-HCl, 5 mM EDTA, 1 mM dithiothreitol and protease inhibitor cocktail, pH 7.4). The cell lysates were homogenized with a 22-gauge needle 10–20 times. The samples were centrifuged at 1,000 x g at 4°C for 10 min to remove nuclei and cell debris. Supernatants were harvested carefully and pellets were discarded. The collected supernatants were fractionized by centrifugation at 100,000 x g at 4°C for 1 h to obtain cell membrane (pellet) and cytosol (supernatant) with a Beckman type 100 Ti rotor (Beckman Coulter). The pellet was resolved with RIPA buffer (25 mM Tris-HCL, pH 7.4, 5 mM EDTA, 137 mM NaCl, 1% Triton X-100, 1% sodium deoxycholate, 0.1% sodium dodecyl sulfate). Total cholesterol was measured using an Amplex Red Cholesterol Assay Kit (Life Technologies) according to the supplier’s instructions.

### Lipid raft fractionation

CHO PS1 WT and ΔE9 cells were washed twice with ice-cold PBS and harvested with trypsin-EDTA. Then, cells were lysed in 4-morpholineethanesulfonic acid (MES)-buffered saline (MBS; 25 mM MES, 150 mM NaCl, pH 6.5) containing 500 mM sodium carbonate with a protease inhibitor cocktail. We also used TEN buffer (25 mM Tris-HCL pH 7.5, 150 mM NaCl, 5 mM EDTA) containing 1% Triton X-100 or 1% Brij-98 with a protease inhibitor cocktail. The lysates were homogenized 20 times with 2 ml homogenizer and homogenized 10 times through a 22-gauge needle. Next, the cell lysates were sonicated for 1 min (20 s sonication followed by 10 s interval). Equal amount of protein was added to 2 ml of 90% (w/v) sucrose in MBS. Then, 4 ml of 35% (w/v) sucrose and 5% (w/v) sucrose in MBS were placed in a 12-ml ultracentrifuge tube to form a discontinuous sucrose gradient. The tubes were placed in a Beckman SW 41 Ti rotor (Beckman Coulter) and centrifuged at 37,500 rpm for 16 h at 4°C. From the top to the bottom, 12 fractions of 1 ml were collected. An equal volume of each fraction was loaded to 10% Tris-Glycine SDS-PAGE gel to detect APP, caveolin, and flotillin. Lipid raft fractions corresponded to lipid raft markers, caveolin, or flotillin. Raft and non-raft fractions were separately combined from 12 fractions and loaded an equal amount of protein to 10% Tris-Glycine SDS-PAGE gel to detect ADAMs (9, 10, 17), BACE, and Nicastrin.

### Western blotting

The proteins were size-fractionated on 10% Tris-Glycine SDS-PAGE gel and transferred to nitrocellulose membrane, and the transferred membrane was blocked with 5% (w/v) non-fat dried milk in Tris-buffered saline with Tween-20 (TBST) for 1 h at room temperature. Then, the blocked membrane was incubated with the following antibodies: anti-CYP51A1 (13431-1-AP, Proteintech), GAPDH (14C10, Cell Signaling Technology), APP (β-Amyloid, 1–16; 303001, BioLegend), ADAM9 (2099, Cell Signaling Technology), ADAM 10 (422751, Calbiochem), ADAM 17 (AB19027, Chemicon), BACE-1 (MAD5308, Millipore), Nicastrin (MAD5556, Chemicon), presenilin 1 (5643, Cell Signaling Technology), caveolin, and flotillin-1 (610059 and 610880, BD Transduction Laboratories) overnight at 4°C. Next, the membrane was washed with TBST four times for 10 min each and incubated with horseradish peroxidase-conjugated goat anti-rabbit IgG or goat anti-mouse IgG antibodies for 2 h at room temperature. After the incubation with the secondary antibody, the membrane was washed again with TBST as described previously. We used enhanced chemiluminescence reagent and a LAS-3000 system (FujiFilm) to detect bands.

### Aβ peptide ELISA assay

CHO PS1 WT and Δ·9 mutant cells were treated with 0, 1, 5, or 10 μM tebuconazole. After 48 h, 1 ml of culture media was collected. The serine protease inhibitor, AEBSF (Sigma-Aldrich) was added to the culture media and centrifuged at 12,000 rpm for 5 min to spin down cell debris. The levels of secreted Aβ40 and Aβ42 were measured using a Human Aβ40 ELISA kit (Invitrogen, KHB3482) and Human Aβ42 ELISA kit (Invitrogen, KHB3442).

### Statistical analysis

Data are expressed as mean ± SEM. We conducted Statistical analysis was carried out using two-way ANOVA and Fisher’s test to determine any interactions between cell types and one way ANOVA between the controls and the treated experimental groups; and considered *P* < 0.05 statistically significant.

## Supporting information

S1 Fig CYP51 expression was increased in CHO PS1 ΔE9 cells compared to PS1 WT cells. (a) Representative western blots show CYP51 (57 kDa) and GAPDH (37 kDa) expression levels from total lysates of CHO PS1 WT and ΔE9 cells. GAPDH was a loading control. (b) Bars correspond to the densitometric analysis of CYP51 expression levels compared to GAPDH expression (n = 6). Student’s t-test: *p<0.05.
(TIF)

S2 Fig Non-ionic detergents might disrupt the localization of APP in lipid rafts. A representative western blot shows APP, caveolin (lipid raft marker), or flotillin (lipid raft marker) expression in the CHO PS1 ΔE9 cells. Cells were homogenized in the presence of non-ionic detergents (a) 1% Triton X-100 or (b) 1% Brij-98. Then, raft and non-raft fractions were obtained using discontinuous sucrose density gradients. When Brij-98 was used, barely detectable level of APP was observed by longer exposure.
(TIF)

S3 Fig The percentage of APP localized in lipid raft fractions was significantly higher in CHO PS1 ΔE9 cells than in PS1 WT cells. The lipid raft (fraction #4 and #5) and non-raft fractions (fractions from #8 to #12) were separately combined for western blotting. Unlike in western blotting experiments from 12 fractions, the equal amount of protein was used for non-raft and raft fraction in these experiments. Caveolin was used as a marker for lipid raft. (a) Representative western blot indicates APP and caveolin. Most of proteins are in non-raft fractions and APP takes part in a small portion of all protein pool. Since the equal amount of proteins was loaded for western blotting, higher APP levels in lipid raft fractions rather than non-raft fractions could be explained. Note that PS1 ΔE9 cells shows significantly reduced APP distribution in non-raft fractions and significantly increased APP localization in raft fractions compared to PS1 WT cells. (b) The densitometric analysis of the percentage of APP levels in raft and non-raft fractions were shown (n = 5, p = 0.01626). Note that the ratio of APP localization in lipid rafts was significantly increased in CHO PS1 ΔE9 cells. Student’s t-test: *p<0.05.
(TIF)

S4 Fig Expression levels of ADAMs, Nicastrin, BACE-1 were not different between the CHO PS1 WT and ΔE9 cells. Raft and non-raft fractions were obtained using discontinuous sucrose density gradients. Raft (fraction #4 and #5) and non-raft (fraction from #8 to #12) fractions were combined. The equal protein concentration of raft and non-raft fractions were loaded for western blotting. (a) A typical western blot showed the levels of ADAM9, ADAM10, ADAM17, Nicastrin, and BACE-1. GAPDH and caveolin-1 were used as markers for non-raft and raft fraction, respectively. Bars correspond to the densitometric analysis of (b) matured-ADAM10, (c) matured-Nicastrin, and (d) BACE-1 (n = 4).
(TIF)

S5 Fig APP localization in lipid rafts was independent of altered γ-secretase activity from CHO PS1 ΔE9 cells. CHO PS1 ΔE9 cells were treated with 500 nM γ-secretase inhibitor IX (Millipore, 565770) for 24 h. Then, raft and non-raft fractions were obtained using discontinuous sucrose density gradient. (a) A representative western blot shows the expression levels of APP and caveolin (lipid rafts marker). (b) The densitometric analysis of the ratio of APP levels in each fraction showed no effect of γ-secretase inhibitor IX (n = 5).
(TIF)

S6 Fig Cholesterol level in CHO PS1 ΔE9 cells was reduced by MβCD. CHO PS1 ΔE9 cells were treated with 0, 2, 5, or 10 mM MβCD for 30 min. Then, membrane and cytosol fractions were obtained. Total membrane cholesterol level was measured with Amplex Red Cholesterol Assay Kit (n = 6). Note that, 5 mM MβCD treatment reduced cholesterol in CHO PS1 ΔE9 cells to a comparable level of PS1 WT cells. Student’s t-test: *p<0.05, **p<0.01, ***p<0.001.
(TIF)

S7 Fig Elevated cholesterol re-localized APP into lipid rafts from CHO PS1 WT cells. CHO PS1 WT cells were treated with 75 μM MβCD-cholesterol for 1.5 h. Raft and non-raft fractions were obtained using discontinuous sucrose density gradient. (a) Representative western blot shows APP and caveolin (lipid rafts marker) from 12 fractions. Levels of APP were increased in lipid raft fractions by MβCD-cholesterol treatment. (b) The densitometric analysis shows that the ratio of APP localized in raft fraction was increased by MβCD-cholesterol (n = 4). Student’s t-test: **p<0.01.
(TIF)

S8 Fig Endogenous APP was not detectable both in lipid raft and non-raft fractions in human neuroblastoma SH-SY5Y cells. A representative western blot shows APP, GAPDH, or caveolin (lipid raft marker) expression in the SH-SY5Y cells. Cells were homogenized with sodium carbonate buffer. Then, raft and non-raft fractions were collected using discontinuous sucrose density gradients. Endogenous APP was barely detectable by longer exposure.
(TIF)

S9 Fig Elevating cholesterol level re-located APP into lipid rafts from SH-SY5Y cells. Cells were stably transfected with APP and BACE-1. Cells were treated with 75 μM MβCD-cholesterol for 30 min. Raft and non-raft fractions were obtained using discontinuous sucrose density gradient. (a) Representative western blot shows the expression levels of APP and flotillin-1 (lipid rafts marker) from 12 fractions. Levels of APP were increased in lipid raft fractions by MβCD-cholesterol treatment. (b) The densitometric analysis shows the ratio of APP localized in raft fraction was increased by MβCD-cholesterol (n = 5). (c) Cholesterol levels (n = 4) and (d) protein levels (n = 4) are shown from sucrose gradient fractions. Student’s t-test: *p<0.05.
(TIF)

S10 Fig CYP51 expression was increased in PS1 ΔE9 cells. (a) Representative western blots show CYP51 and GAPDH expression levels from total lysates of CHO PS1 WT and PS1 ΔE9 cells. Cells were pretreated with indicated concentrations of tebuconazole for 48 h. GAPDH was a loading control. (b) Bars correspond to the densitometric analysis of CYP51 expression levels compared to GAPDH expression (n = 5). Student’s t-test: *p<0.05, **p<0.01.
(TIF)

S11 Fig The localization of APP in lipid rafts was not changed by tebuconazole in CHO PS1 WT cells. CHO PS1 WT cells were treated with 10 μM tebuconazole for 48 h. Using discontinuous sucrose density gradient, raft and non-raft fractions were obtained. (a) Representative western blot indicated APP and caveolin (lipid rafts marker) from each fraction. Levels of APP in lipid raft fractions were not altered by tebuconazole treatment. (b) The densitometric analysis of the ratio of APP levels in each fraction was shown. (n = 4). (c) Analysis of cholesterol levels from each fraction (n = 4).
(TIF)

S12 Fig Levels of presenilin expression were not different between the CHO PS1 WT and ΔE9 cells. (a) Representative western blots showed full length presenilin 1 and GAPDH (loading control) from total lysates of CHO PS1 WT and ΔE9 cells. (b) Bars correspond to the densitometric analysis of full length presenilin 1 (n = 4).
(TIF)
