# Supplementary figures and images for: Elevated cellular cholesterol in Familial Alzheimer’s presenilin 1 mutation is associated with lipid raft localization of β-amyloid precursor protein
Source: PLoS One. 2019 Jan 25;14(1):e0210535. doi: 10.1371/journal.pone.0210535 (PMC6347419; doi:10.1371/journal.pone.0210535)

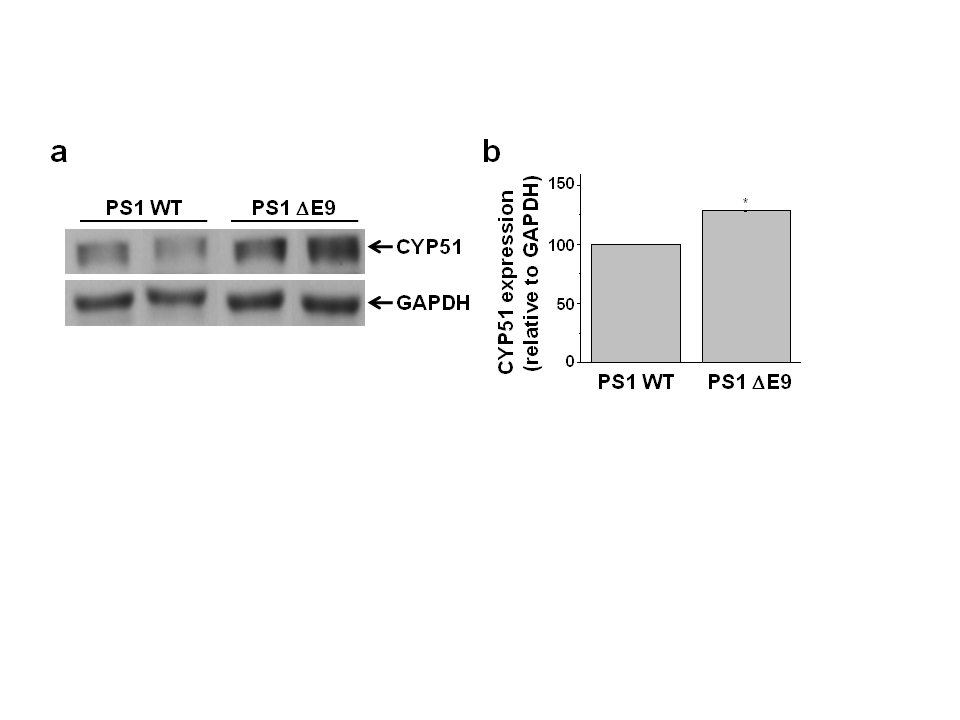

Supplement: S1 Fig — (a) Representative western blots show CYP51 (57 kDa) and GAPDH (37 kDa) expression levels from total lysates of CHO PS1 WT and ΔE9 cells. GAPDH was a loading control. (b) Bars correspond to the densitometric analysis of CYP51 expression levels compared to GAPDH expression (n = 6). Student’s t-test: *p<0.05. (TIF) [file pone.0210535.s001.TIF]

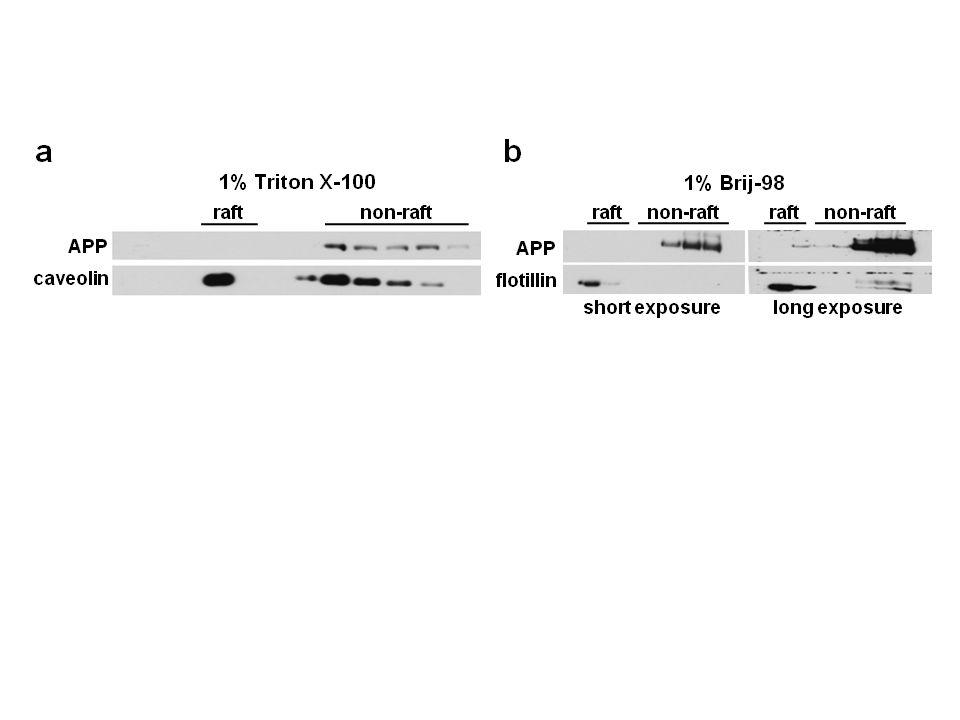

Supplement: S2 Fig — A representative western blot shows APP, caveolin (lipid raft marker), or flotillin (lipid raft marker) expression in the CHO PS1 ΔE9 cells. Cells were homogenized in the presence of non-ionic detergents (a) 1% Triton X-100 or (b) 1% Brij-98. Then, raft and non-raft fractions were obtained using discontinuous sucrose density gradients. When Brij-98 was used, barely detectable level of APP was observed by longer exposure. (TIF) [file pone.0210535.s002.TIF]

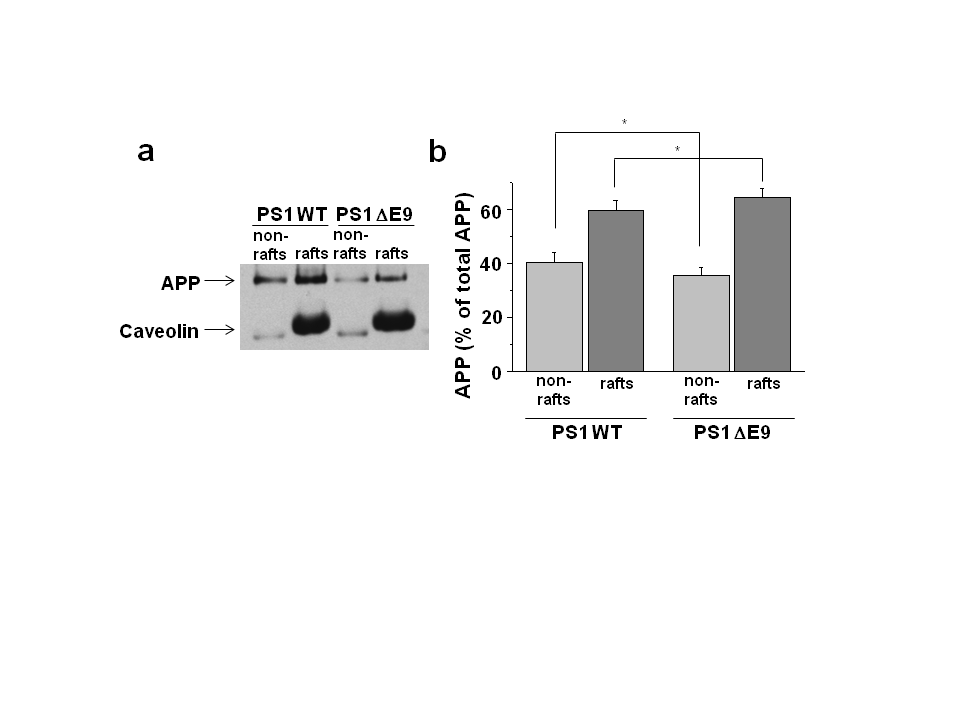

Supplement: S3 Fig — The lipid raft (fraction #4 and #5) and non-raft fractions (fractions from #8 to #12) were separately combined for western blotting. Unlike in western blotting experiments from 12 fractions, the equal amount of protein was used for non-raft and raft fraction in these experiments. Caveolin was used as a marker for lipid raft. (a) Representative western blot indicates APP and caveolin. Most of proteins are in non-raft fractions and APP takes part in a small portion of all protein pool. Since the equal amount of proteins was loaded for western blotting, higher APP levels in lipid raft fractions rather than non-raft fractions could be explained. Note that PS1 ΔE9 cells shows significantly reduced APP distribution in non-raft fractions and significantly increased APP localization in raft fractions compared to PS1 WT cells. (b) The densitometric analysis of the percentage of APP levels in raft and non-raft fractions were shown (n = 5, p = 0.01626). Note that the ratio of APP localization in lipid rafts was significantly increased in CHO PS1 ΔE9 cells. Student’s t-test: *p<0.05. (TIF) [file pone.0210535.s003.TIF]

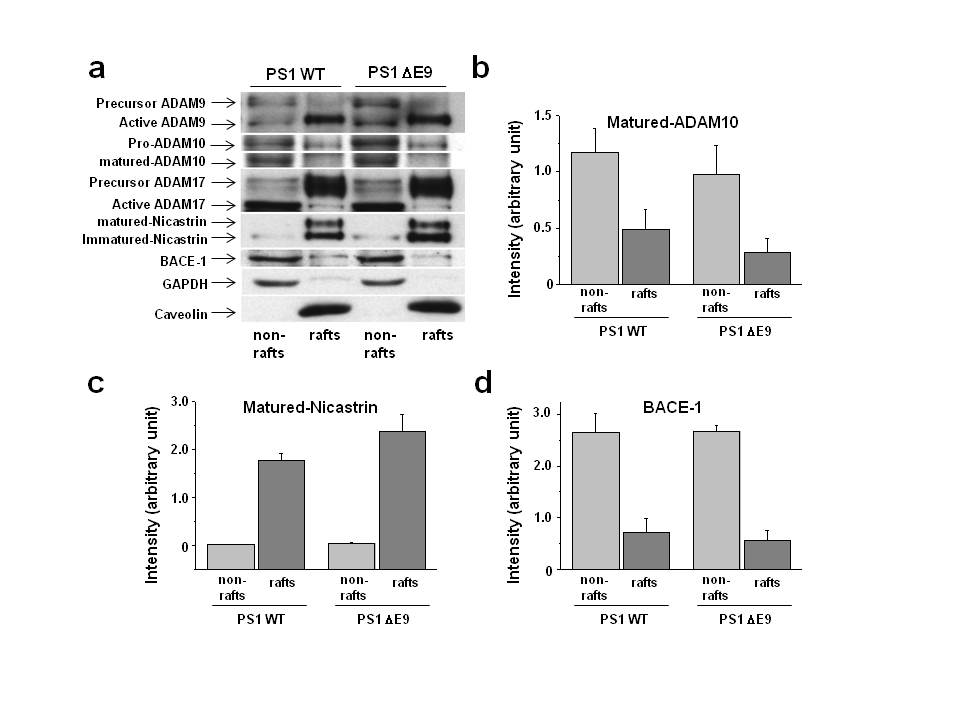

Supplement: S4 Fig — Raft and non-raft fractions were obtained using discontinuous sucrose density gradients. Raft (fraction #4 and #5) and non-raft (fraction from #8 to #12) fractions were combined. The equal protein concentration of raft and non-raft fractions were loaded for western blotting. (a) A typical western blot showed the levels of ADAM9, ADAM10, ADAM17, Nicastrin, and BACE-1. GAPDH and caveolin-1 were used as markers for non-raft and raft fraction, respectively. Bars correspond to the densitometric analysis of (b) matured-ADAM10, (c) matured-Nicastrin, and (d) BACE-1 (n = 4). (TIF) [file pone.0210535.s004.TIF]

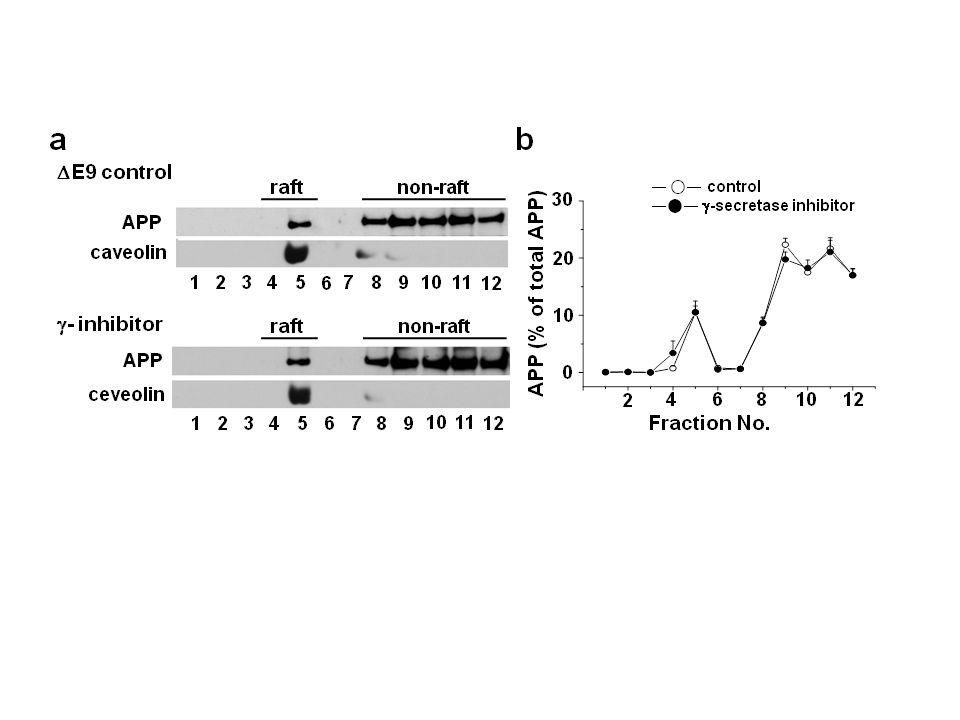

Supplement: S5 Fig — CHO PS1 ΔE9 cells were treated with 500 nM γ-secretase inhibitor IX (Millipore, 565770) for 24 h. Then, raft and non-raft fractions were obtained using discontinuous sucrose density gradient. (a) A representative western blot shows the expression levels of APP and caveolin (lipid rafts marker). (b) The densitometric analysis of the ratio of APP levels in each fraction showed no effect of γ-secretase inhibitor IX (n = 5). (TIF) [file pone.0210535.s005.TIF]

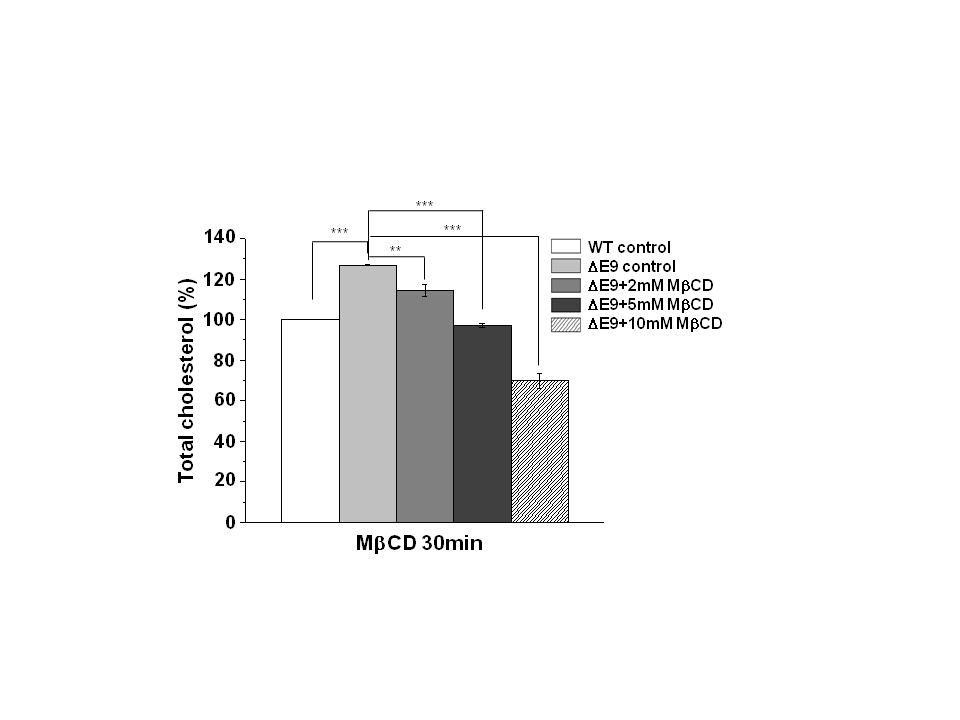

Supplement: S6 Fig — CHO PS1 ΔE9 cells were treated with 0, 2, 5, or 10 mM MβCD for 30 min. Then, membrane and cytosol fractions were obtained. Total membrane cholesterol level was measured with Amplex Red Cholesterol Assay Kit (n = 6). Note that, 5 mM MβCD treatment reduced cholesterol in CHO PS1 ΔE9 cells to a comparable level of PS1 WT cells. Student’s t-test: *p<0.05, **p<0.01, ***p<0.001. (TIF) [file pone.0210535.s006.TIF]

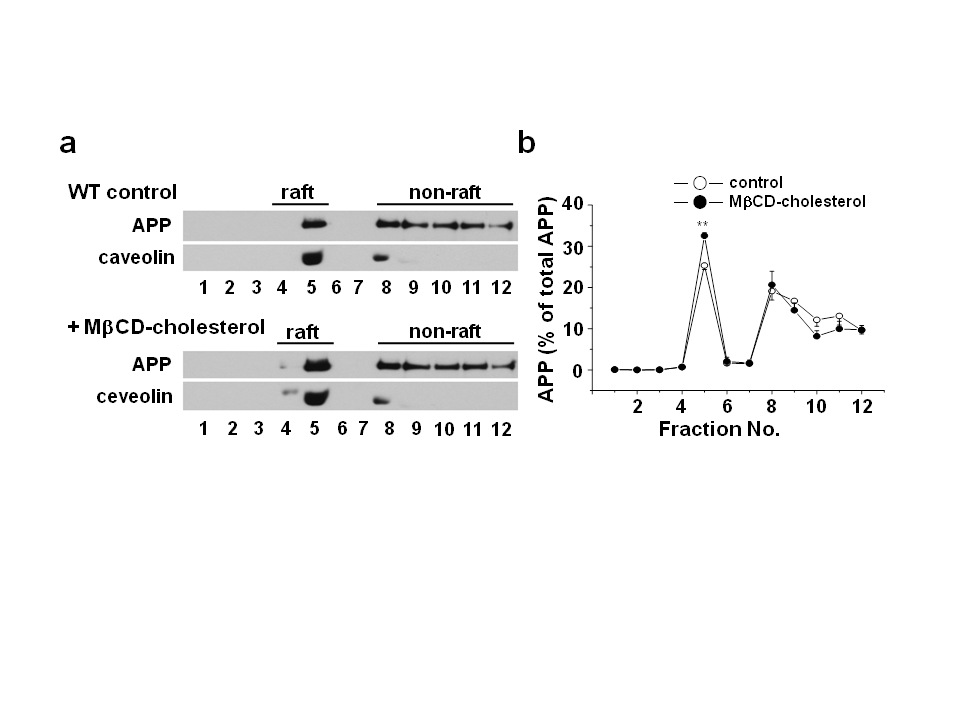

Supplement: S7 Fig — CHO PS1 WT cells were treated with 75 μM MβCD-cholesterol for 1.5 h. Raft and non-raft fractions were obtained using discontinuous sucrose density gradient. (a) Representative western blot shows APP and caveolin (lipid rafts marker) from 12 fractions. Levels of APP were increased in lipid raft fractions by MβCD-cholesterol treatment. (b) The densitometric analysis shows that the ratio of APP localized in raft fraction was increased by MβCD-cholesterol (n = 4). Student’s t-test: **p<0.01. (TIF) [file pone.0210535.s007.TIF]

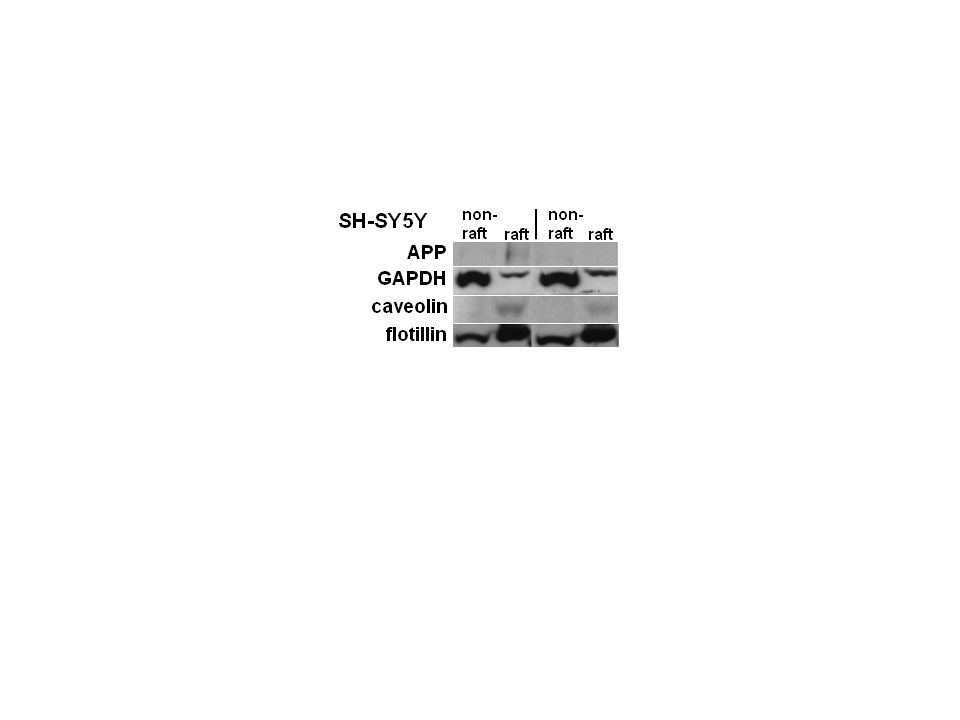

Supplement: S8 Fig — A representative western blot shows APP, GAPDH, or caveolin (lipid raft marker) expression in the SH-SY5Y cells. Cells were homogenized with sodium carbonate buffer. Then, raft and non-raft fractions were collected using discontinuous sucrose density gradients. Endogenous APP was barely detectable by longer exposure. (TIF) [file pone.0210535.s008.TIF]

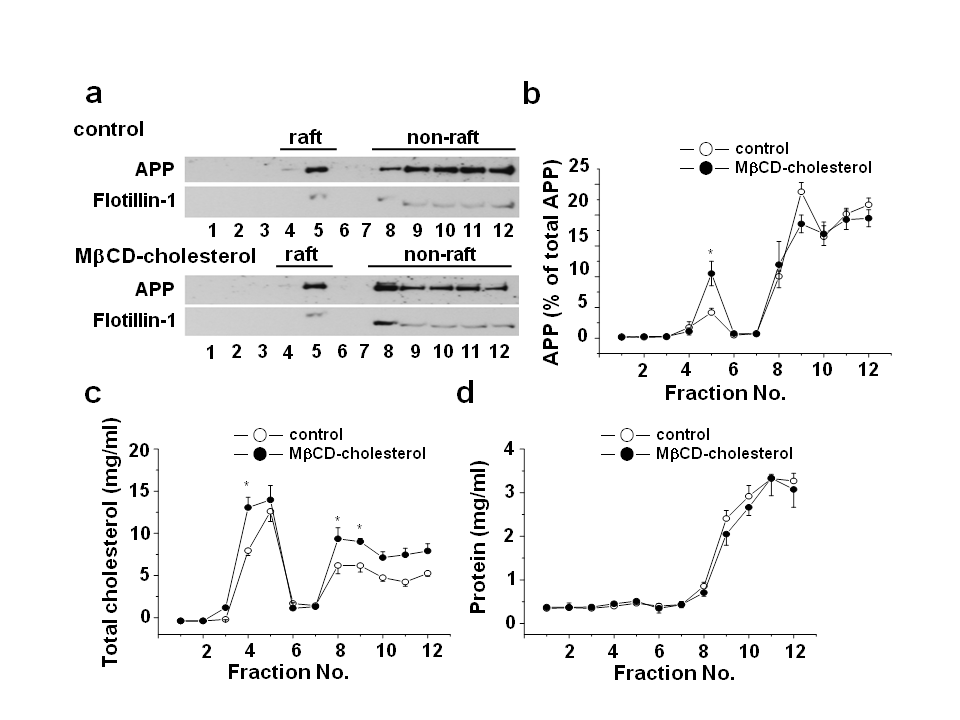

Supplement: S9 Fig — Cells were stably transfected with APP and BACE-1. Cells were treated with 75 μM MβCD-cholesterol for 30 min. Raft and non-raft fractions were obtained using discontinuous sucrose density gradient. (a) Representative western blot shows the expression levels of APP and flotillin-1 (lipid rafts marker) from 12 fractions. Levels of APP were increased in lipid raft fractions by MβCD-cholesterol treatment. (b) The densitometric analysis shows the ratio of APP localized in raft fraction was increased by MβCD-cholesterol (n = 5). (c) Cholesterol levels (n = 4) and (d) protein levels (n = 4) are shown from sucrose gradient fractions. Student’s t-test: *p<0.05. (TIF) [file pone.0210535.s009.TIF]

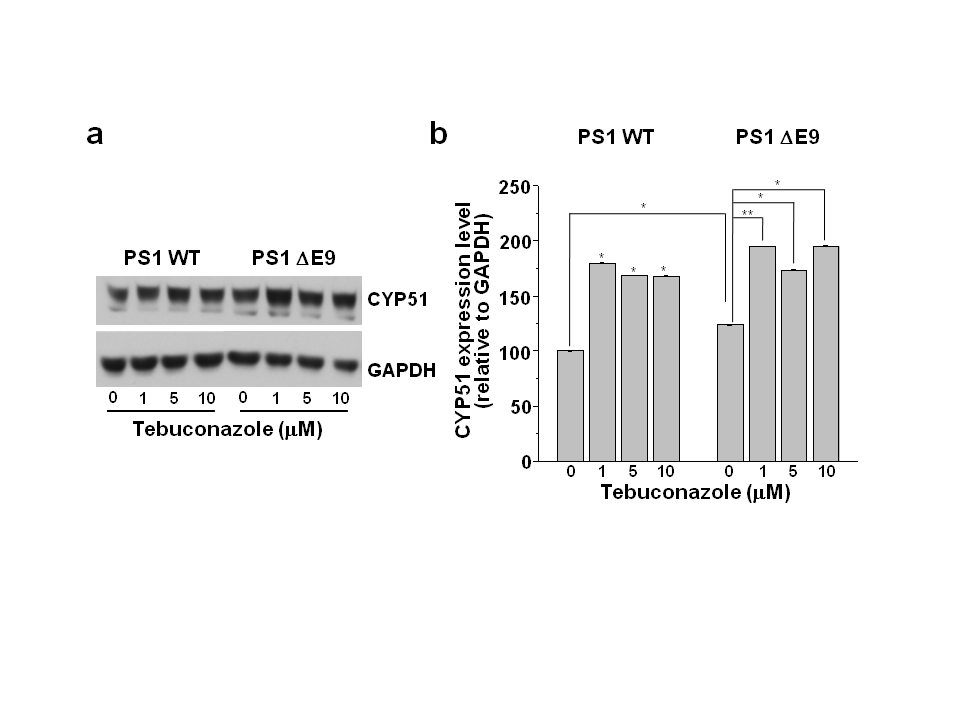

Supplement: S10 Fig — (a) Representative western blots show CYP51 and GAPDH expression levels from total lysates of CHO PS1 WT and PS1 ΔE9 cells. Cells were pretreated with indicated concentrations of tebuconazole for 48 h. GAPDH was a loading control. (b) Bars correspond to the densitometric analysis of CYP51 expression levels compared to GAPDH expression (n = 5). Student’s t-test: *p<0.05, **p<0.01. (TIF) [file pone.0210535.s010.TIF]

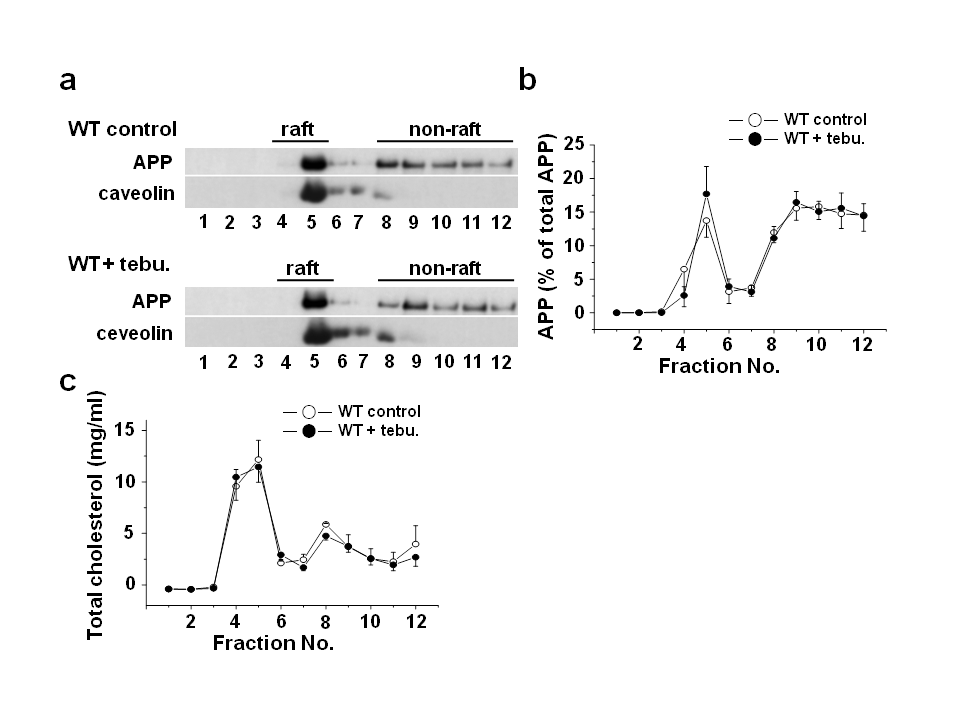

Supplement: S11 Fig — CHO PS1 WT cells were treated with 10 μM tebuconazole for 48 h. Using discontinuous sucrose density gradient, raft and non-raft fractions were obtained. (a) Representative western blot indicated APP and caveolin (lipid rafts marker) from each fraction. Levels of APP in lipid raft fractions were not altered by tebuconazole treatment. (b) The densitometric analysis of the ratio of APP levels in each fraction was shown. (n = 4). (c) Analysis of cholesterol levels from each fraction (n = 4). (TIF) [file pone.0210535.s011.TIF]

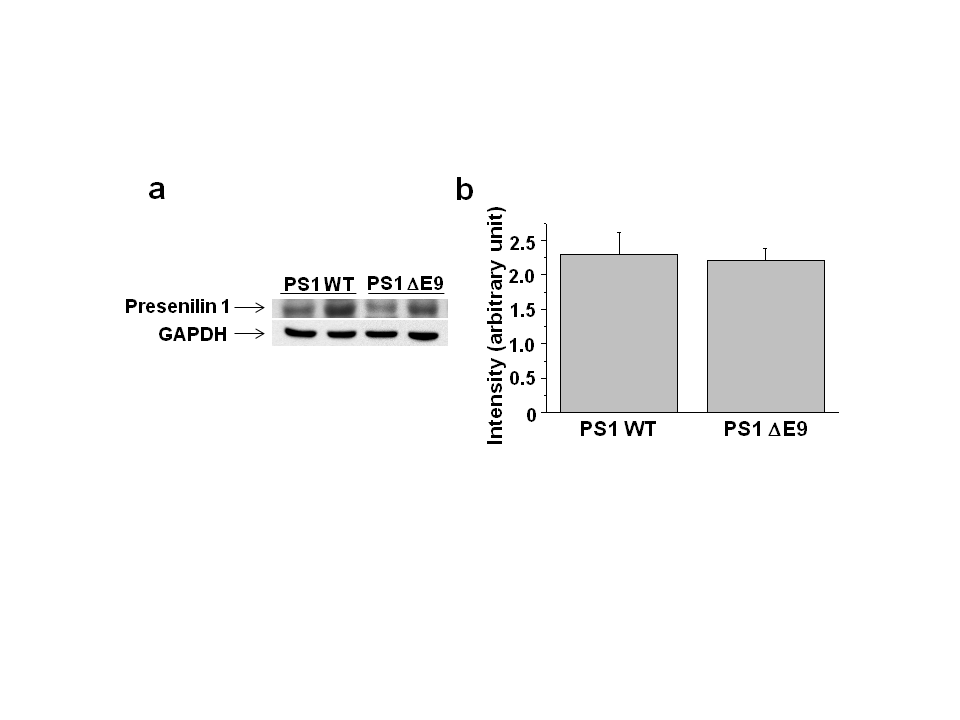

Supplement: S12 Fig — (a) Representative western blots showed full length presenilin 1 and GAPDH (loading control) from total lysates of CHO PS1 WT and ΔE9 cells. (b) Bars correspond to the densitometric analysis of full length presenilin 1 (n = 4). (TIF) [file pone.0210535.s012.TIF]
